# Fluorescent Protein Based FRET Pairs with Improved Dynamic Range for Fluorescence Lifetime Measurements

**DOI:** 10.1371/journal.pone.0134436

**Published:** 2015-08-03

**Authors:** Bobin George Abraham, Karen S. Sarkisyan, Alexander S. Mishin, Ville Santala, Nikolai V. Tkachenko, Matti Karp

**Affiliations:** 1 Department of Chemistry and Bioengineering, Tampere University of Technology, P.O. Box 541, 33101, Tampere, Finland; 2 Shemyakin-Ovchinnikov Institute of Bioorganic Chemistry, Miklukho-Maklaya 16/10, 117997, Moscow, Russia; The Beatson Institute for Cancer Research, UNITED KINGDOM

## Abstract

Fluorescence Resonance Energy Transfer (FRET) using fluorescent protein variants is widely used to study biochemical processes in living cells. FRET detection by fluorescence lifetime measurements is the most direct and robust method to measure FRET. The traditional cyan-yellow fluorescent protein based FRET pairs are getting replaced by green-red fluorescent protein variants. The green-red pair enables excitation at a longer wavelength which reduces cellular autofluorescence and phototoxicity while monitoring FRET. Despite the advances in FRET based sensors, the low FRET efficiency and dynamic range still complicates their use in cell biology and high throughput screening. In this paper, we utilized the higher lifetime of NowGFP and screened red fluorescent protein variants to develop FRET pairs with high dynamic range and FRET efficiency. The FRET variations were analyzed by proteolytic activity and detected by steady-state and time-resolved measurements. Based on the results, NowGFP-tdTomato and NowGFP-mRuby2 have shown high potentials as FRET pairs with large fluorescence lifetime dynamic range. The *in vitro* measurements revealed that the NowGFP-tdTomato has the highest Förster radius for any fluorescent protein based FRET pairs yet used in biological studies. The developed FRET pairs will be useful for designing FRET based sensors and studies employing Fluorescence Lifetime Imaging Microscopy (FLIM).

## Introduction

Fluorescence Resonance Energy Transfer (FRET) between fluorescent proteins has become a popular tool to study the protein localization and biochemical conditions inside a living cell. Conventionally, Cyan and Yellow variants of fluorescent proteins are widely used for FRET studies. With the development of Orange and Red variants of Fluorescent proteins, the Cyan-Yellow FRET pairs are getting replaced as longer wavelength excitation and emission results in reduced cellular autofluorescence, decreased phototoxicity and lower light scattering [1,2]. Furthermore, the Cyan and Yellow FRET pair exhibits noticeable crosstalk which results in problematic spectral separation and low FRET dynamic range (donor/acceptor emission ratio variation) limits its application when the intracellular responses are subtle or transient [2,3]. Circularly permuted fluorescent protein variants [4] and optimizing the linkers [5,6] improves FRET dynamic range to an extent, but use of new improved fluorescent proteins as FRET pairs is required to evade the drawbacks of current FRET based sensors.

The detection method of FRET is also crucial to obtain accurate information. FRET can be detected by intensity and fluorescence lifetime based methods [7,8]. Intensity based detection methods are sensitive to variations in probe concentration and optical path length [9,10]. In addition, calibration and correction procedures are needed to overcome the concentration changes, spectral bleed through, back-bleed through and photobleaching [11–14]. The fluorescence lifetime based method is independent of probe concentration and optical pathlength [15]. It is less susceptible to sample absorbance, scattering, spectral bleed through and photobleaching making it one of the most direct method to measure FRET [10,16]. Taking all this together, fluorescence lifetime imaging microscopy (FLIM) is a superior method to determine FRET in cell biology. For lifetime measurements using fluorescent proteins, the typical lifetime of fluorescent proteins range from 1.9 to 4 ns. Utilizing fluorescent proteins with long lifetime could improve the dynamic range of FRET sensors in FLIM [15]. NowGFP is an improved version of green fluorescent protein WasCFP with tryptophan-based chromophore in anionic state and a fluorescence lifetime of ~5 ns. This is the longest fluorescence lifetime reported for any green fluorescent protein variant [17]. In intracellular conditions, the lifetime of NowGFP was reduced to 4–4.5 ns. Despite this reduction, it is the longest lifetime reported for a green variant of GFP and can be imaged simultaneously with another GFP with high lifetime contrast [18]. Besides the longest lifetime, this fluorescent protein variant is 30% brighter than EGFP and has a molar extinction coefficient of 56700 M^−1^cm^−1^ with a high emission quantum yield (0.76) making it an excellent potential donor for FRET and FLIM [18].

In this study, we exploited these superior properties of NowGFP to develop novel FRET pairs with high dynamic range. The FRET between NowGFP and red fluorescent protein (RFP) variants was studied by generating FRET pairs fused with a linker comprising thrombin protease cleavage site. The FRET was studied using steady-state, time-resolved single photon counting (TCSPC) and FLIM spectroscopy techniques.

## Materials and Methods

### Construction of FRET pairs

Molecular biology techniques were performed as described [19]. The FRET pairs were created by cloning thrombin protease cleavage site and red fluorescent protein variants to NowGFP/pQE-30 vector generating NowGFP-GGGSLVPRGS-RFP variant FRET pair. The amino acid sequence of NowGFP is provided in (S1 Fig). The red fluorescent protein variants used for this study are mRuby2 (Addgene plasmid 49089, deposited by Kurt Beam), mOrange (Addgene plasmid 29770, deposited by Scott Gradia), tdTomato (Addgene plasmid 18879, deposited by Robert Campbell [20] and TagRFP (Evrogen). Thrombin cleavage site (LVPR) was flanked by aminoacid linkers GGGS and GS at N-terminal and C-terminal, respectively and the whole region was synthesized by adding the sequence to the primer used for amplifying NowGFP from the NowGFP-pQE-30 vector. The red fluorescent proteins variants were PCR-amplified and the amplified product comprise overlapping region of thrombin cleavage site at the 5’ end and *Hind*III site at the 3’ end. The overlapping region and the restriction site were generated by adding the corresponding sequences to the primers. The whole sequence for the FRET constructs was then generated by overlap extension PCR and the products were cloned to pQE-30 vector using *EcoR*I/*Hind*III site. The primer sequences and PCR details are provided in S1 and S2 Tables. The final constructs obtained are: NowGFP-GGGSLVPRGS-mRuby2 (NowGFP-mRuby2), NowGFP-GGGSLVPRGS-mOrange (NowGFP-mOrange), NowGFP-GGGSLVPRGS-tdTomato (NowGFP-tdTomato) and NowGFP-GGGSLVPRGS-TagRFP (NowGFP-TagRFP). Constructs with single fluorescent proteins were also made by cloning the PCR-amplified product corresponding fluorescent protein to pQE-30 vector using *BamH*1/*Hind*III sites. A control construct with NowGFP-mRuby2 FRET pair was made and this construct lacks thrombin cleavage site (Instead of LVPR, the construct has LVPS). The constructs were introduced into *Escherichia coli* XL1-Blue (Stratagene, USA) cells by electroporation. All plasmids were verified by sequencing.

### Protein production and characterization

For protein production, the cells were cultivated in low-salt LB medium (10 g/L tryptone, 5 g/L yeast extract, 5 g/L NaCl, pH 7.0) with appropriate antibiotics at 37°C and 300 rpm in fermentor (1 L) (Biostat-B plus, Sartorius BBI systems, GmbH). The antibiotics used were 50 μg/mL of ampicillin (for cells with pQE-30 vector) and 25 μg/mL of chloramphenicol (for cells with pAK400c vector). Protein production and purification was performed as described previously [21]. The purified protein was buffer exchanged with NAP columns (Sephadex G-25, Novagen) into cleavage buffer containing 10 mM Tris (pH 8.0), 100 mM NaCl and 1 mM EDTA.

### Proteolytic activity and *in vitro* fluorescence spectroscopy

The proteolytic activity was analyzed by addition of 10 NIH units of Thrombin (Sigma, USA) to the FRET constructs which was buffer exchanged to cleavage buffer. Fluorescence emission measurements were taken at different time intervals for one hour. Fluorescence measurements were carried out by Fluorometer Fluorolog-3-111 (ISA-JobinYvon, France). The emission spectra were corrected using the correction function supplied by the manufacturer after subtracting dark counts of photomultiplier. A portion of the cleaved FRET construct as well as untreated FRET construct was loaded to SDS-PAGE (Amersham ECL Gel 10%- GE Health care Life sciences) to analyze the proteolytic activity. The SDS-Gel was stained using PageBlue Protein Staining Solution (Thermo Scientific, USA) to observe the proteins.

Fluorescence lifetime was measured using time-correlated single photon counting (TCSPC) technique. The TCSPC instrument used consisted of pulsed laser diode (LDH-P-C-485, PicoQuant Germany), cooled multichannel Photon Multiplying Tube (R3809U-50,Hamamatsu, Japan), and TCSPC module (PicoHarp 300, PicoQuant Germany), which combine constant fraction discriminators, time-to-amplitude converter (TAC), and multichannel analyzer (MCA) (PicoQuant, Germany). The samples were excited at 483 nm using LDH-P-C-485 pulsed laser diode with a pulse repetition rate of 2.5 MHz. The fluorescence decay was collected until 10000 counts accumulated at maximum with a time resolution of 16 ps and instrument response function (fwhm) ≈ 100 ps. The emission was monitored at 515 nm where only the donor has the emission and cut off filters were used to prevent the excitation source to reach the photomultiplier tube.

For photobleaching experiments, NowGFP and EGFP (in pQE-30 plasmid) expressing *E*. *coli* cells in Phosphate-buffered saline (pH-7.4) was imaged with Leica confocal inverted microscope DMIRE2 TCS SP2 (Leica, Wetzlar, Germany) equipped with 63x oil objective. 488 nm laser was used for excitation and the laser power was set to be either 141 μW or 23 μW. To measure the photobleaching rate we took series of 512 × 512 pixels images with 400 Hz scanning speed.

### FLIM analysis

For FILM, *E*. *coli* cells expressing the fluorescent proteins were placed on a microscope cover slip coated with 2% agarose gel in cleavage buffer. A second cover slip was placed after the addition of cells to obtain uniform surface and to fix the cells. The single cell analysis was performed using Fluorescence Lifetime Microscope (FLM) MicroTime 200 (PicoQuant) with a 100× (1.49NA, oil) objective. The laser diode LDH-P-C483 (PicoQuant) emitting at 483 nm were used for excitation and the emission was monitored using 510/20 nm detection filter. The images were acquired with scan steps of 0.1 μm, time interval of 5 ms per pixel and the total scan area of 20 × 20 μm^2^. FRET was analyzed by monitoring the fluorescence lifetime of the donor in the presence and absence of acceptor.

### Data Analysis

The Förster distance (*R*_*0*_) of the FRET pairs was calculated from the molar absorption and emission spectra of the single fluorescent proteins using Eq 1 [22]

$$R_{0}^{6}=8.785\times 10^{2}\kappa^{2}Q_{D}n^{-4}J\left(\lambda \right)$$ (1)

where κ^2^ is the orientation factor between the FRET pair, *Q*_*D*_ is the emission quantum yield of the donor, *n* is the refractive index of the medium. *κ* ^2^ is taken as 2/3 expecting random orientation of the fluorophores and *n* is taken as 1.33 (n of water). *J*(*λ*) is the spectral overlap integral and it is given by Eq 2 [22]

$$J\left(\lambda \right)=\int \;F_{D}\;\left(\lambda \right)\varepsilon_{A}\left(\lambda \right)\lambda^{4}d\lambda$$ (2)

where *F*_*D*_ is the peak-normalized donor fluorescence spectrum, *ε*_*A*_ is the molar absorption spectrum of the acceptor, both as a function of wavelength (λ). The *R*_*0*_ value is in units of angstroms, while *ε*_*A*_ is in M^-1^cm^-1^ and λ is in nanometers.

For TCSPC measurements, the emission decay curves were fitted using deconvolution with the instrument response function and applying mono and biexponential decay models to obtain the lifetime. The calculations were carried out using in-house software (DecFit). For intracellular measurements using fluorescence lifetime microscope MicroTime 200 (PicoQuant), the image analysis and curve fitting was performed using the supplied SymPhoTimev. 4.7 software. FRET efficiency was calculated from the results obtained by the curve fitting using Eq 3 [23]

$$E=1-\left(\tau_{DA}/\tau_{D}\right)$$ (3)

where τ_DA_ and τ_D_ are the fluorescence lifetimes of the donor in the presence and absence of acceptor.

## Results

### Evaluation of the FRET pairs

**Fig 1:**
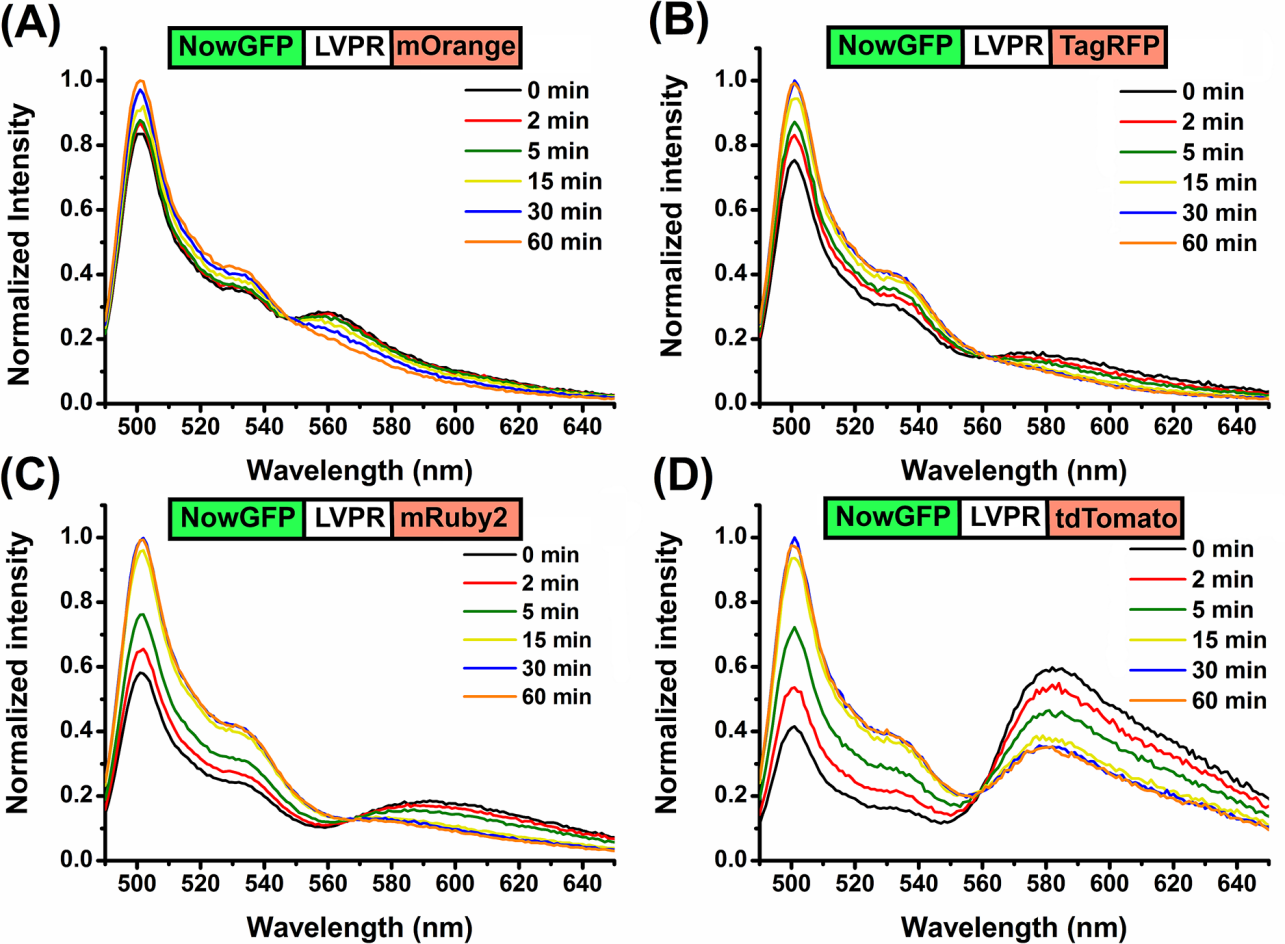
Emission spectra of the FRET constructs. Fluorescence emission spectrum (at 480 nm excitation) of the FRET constructs treated with thrombin. The schematics of the FRET constructs are displayed above the spectrum. "LVPR" represents the sequence GGGSLVPRGS. The decrease in the FRET in time as a result of proteolytic cleavage can be observed from the spectrum.

**Fig 2:**
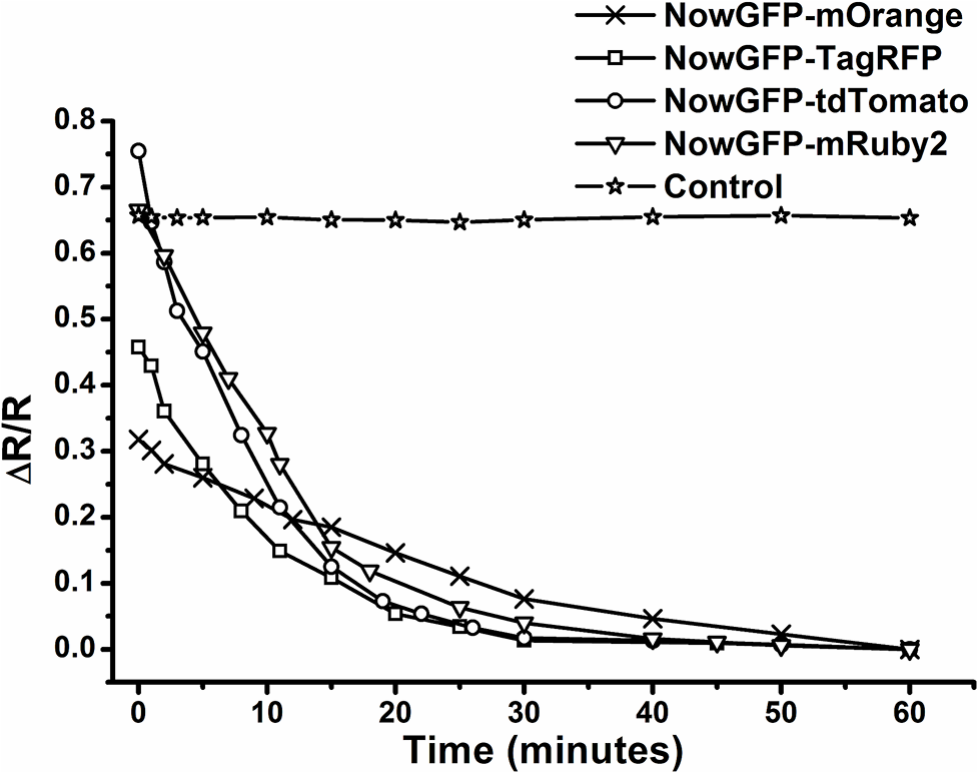
Variations in the FRET response of FRET pairs on proteolytic cleavage over time. The control is NowGFP-mRuby2 FRET pair without thrombin cleavage site (LVPS instead of LVPR). ΔR/R was computed as (R_0_-R_F_)/R_0_ where R is donor:acceptor ratio and R_0_ is the donor:acceptor ratio when there is no FRET and R_F_ is the FRET ratio

The fluorescence spectrum was used to analyze the spectral overlap integral (*J*(*λ*)) and the Förster radius (*R*_0_) of the FRET pairs. The fluorescence properties of the proteins used in this study along with the *R*_0_ and *J*(*λ*) values are provided in Table 1. Among the four red fluorescent protein variants studied, NowGFP-tdTomato and NowGFP-mRuby2 FRET pair demonstrates higher spectral overlap and *R*_0_. The *R*_0_ of these two FRET pairs is greater than the *R*_0_ of the other fluorescent protein based FRET pairs [2,24,25]. The larger *R*_0_ value indicates the higher probability of the two FRET pairs to be used for developing superior FRET based sensors with high dynamic range. The correlation between higher *R*_0_ and FRET was verified from the steady-state spectroscopy data which displayed higher FRET for NowGFP-tdTomato followed by NowGFP-mRuby2 and comparatively lower FRET for NowGFP-TagRFP and NowGFP-mOrange. For FRET analysis, we utilized the thrombin recognition sequence (LVPR) incorporated between the FRET pairs. When the fusion proteins was excited at 483 nm, where only the donor (NowGFP) is excited, an enhanced fluorescence emission was observed between 560 nm and 610 nm for the different FRET pairs indicating energy transfer (Fig 1). After addition of thrombin, an increase in donor emission was observed (at 515 nm) with a decrease in the emission of acceptors (between 560 and 610 nm) diminishing FRET. The reduction in FRET indicates the cleavage of the thrombin site resulting in the separation of FRET pairs. The cleavage of protein started immediately after the addition of thrombin and the change in FRET response indicated more than 90% of protein cleavage within 30 minutes after addition of thrombin (Fig 2). There was no further change in FRET response after 60 minutes due to the complete cleavage of the proteins. The control FRET construct, which lacks thrombin cleavage site, showed no response on protease treatment and this confirms that the change in FRET is due to the site-specific cleavage by thrombin resulting in the separation of the FRET pairs.

**Table 1:** Spectral properties of the fluorescent proteins along with the Förster radius of the FRET pairs.

Fluorescent protein	λ_Abs_ (nm)	λ_Emiss_ (nm)	ε (M^- 1^ cm^- 1^)	QY	J (*λ*)M^-1^cm^-1^nm^4^ *	R_0_ (Å)*
NowGFP	494	502	56700	0.76	-	
mOrange	548	562	71000†	0.69†	2.48 × 10^15^	57.63
mRuby2	559	600	113000‡	0.38‡	3.74 × 10^15^	61.72
TagRFP	555	584	100000§	0.48§	2.91 × 10^15^	59.17
tdTomato	554	581	138000†	0.69†	5.43 × 10^15^	65.67

* With NowGFP as donor (This study)

† Values from ref. [33]

‡ Values from ref. [2]

§ Values from ref. [46]

**Fig 3:**
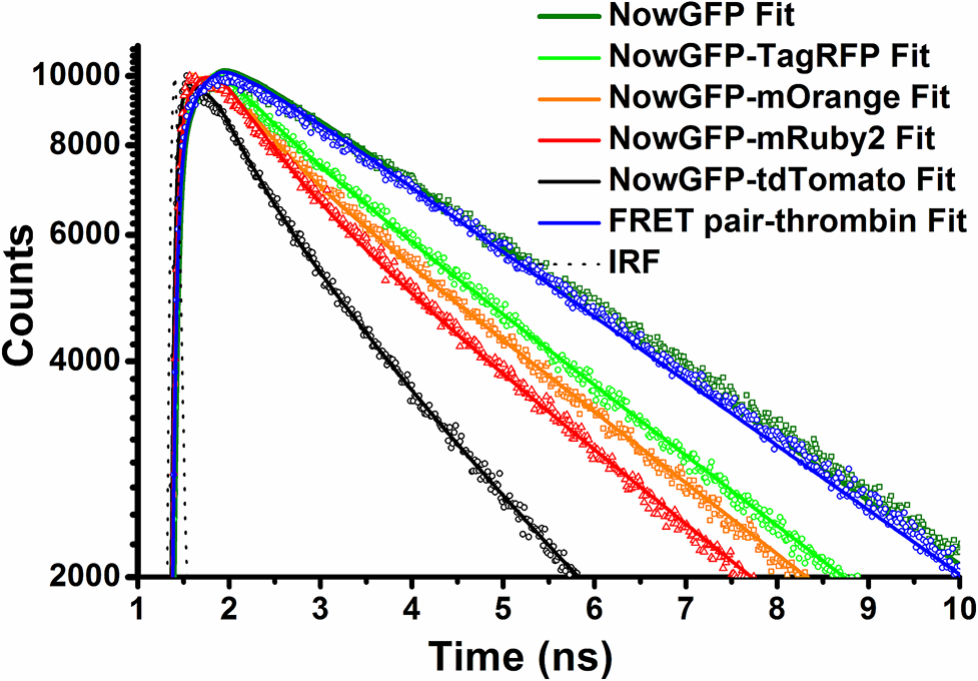
Fluorescence decay and fits. Fluorescence decay and fits of the FRET pairs at monitoring wavelength of 515 ns. At this monitoring wavelength only the donor decay can be observed. NowGFP Fit denotes the lifetime decay of the donor alone and FRET pair-thrombin Fit denotes the lifetime of the donor after proteolytic cleavage. The quenching of the fluorescence lifetime as a result of FRET can be observed from the decay of the FRET pairs. The lines indicate the fit and the symbols to the corresponding color of the line indicate the decay curve. The full time window of the decay and fits along with the residuals of the fits is shown in S2 Fig.

As this study aims to utilize the higher fluorescence lifetime of NowGFP for generating novel FRET pairs, fluorescence lifetime of the purified FRET constructs was analyzed by the TCSPC technique. The samples were excited at 483 nm and the fluorescence decays were monitored at 515 nm. At this monitoring wavelength, only the donor emits and none of the acceptors has noticeable emission which makes possible to determine the donor excited state lifetime for all the studied pairs. The single exponential decay of non-fused NowGFP showed very long lifetime of 5.00 ns ± 0.03 ns, which was similar to the previously reported NowGFP lifetime [18]. The fluorescence lifetime of NowGFP in the FRET pairs after thrombin treatment displayed lifetime of ~4.8–5.0 ns for different FRET pairs. This indicates that the treatment by thrombin has resulted in the complete cleavage of the FRET pairs diminishing FRET and restoring the original lifetime of NowGFP. The fluorescence decay curve of the fused FRET constructs showed large decrease in the fluorescence lifetime of donor confirming the efficient energy transfer (Fig 3). For the FRET constructs undergoing FRET, bi-exponential fitting models were used to extract the fluorescence lifetimes. The average weighted fluorescence lifetime of the FRET constructs (Table 2) showed higher dynamic range for NowGFP-tdTomato and NowGFP-mRuby2 FRET pairs.

**Table 2:** Fluorescence lifetime and FRET efficiency of the FRET pairs *in vitro*.

Fluorescent protein/pairs	τ_ave_	χ^2^	E
NowGFP (Donor alone)	5.00 ± 0.03	1.42	-
NowGFP-mOrange	3.42 ± 0.05	1.18	0.30
NowGFP-TagRFP	3.74 ± 0.07	1.13	0.25
NowGFP-mRuby2	2.84 ± 0.05	1.20	0.43
NowGFP-tdTomato	2.02 ± 0.02	1.01	0.59
FRET pairs after proteolytic cleavage*	4.88 ± 0.02	1.37	-

* Average from all the FRET pairs after thrombin treatment. E—FRET efficiency (calculated according to Eq 3). χ2—calculated standard weighted least squares to assess the goodness of the fit

Determined from the fluorescence lifetime data, the FRET efficiency, *E*, of NowGFP-tdTomato was highest (0.59) (Table 2). It should be noted that this high FRET efficiency can vary based on the linker size and flexibility, and based on the dipole orientation [26]. Based on the results from the steady-state measurement and fluorescence lifetime measurements NowGFP-tdTomato and NowGFP-mRuby were selected for further studies such as FLIM analysis.

**Fig 4:**
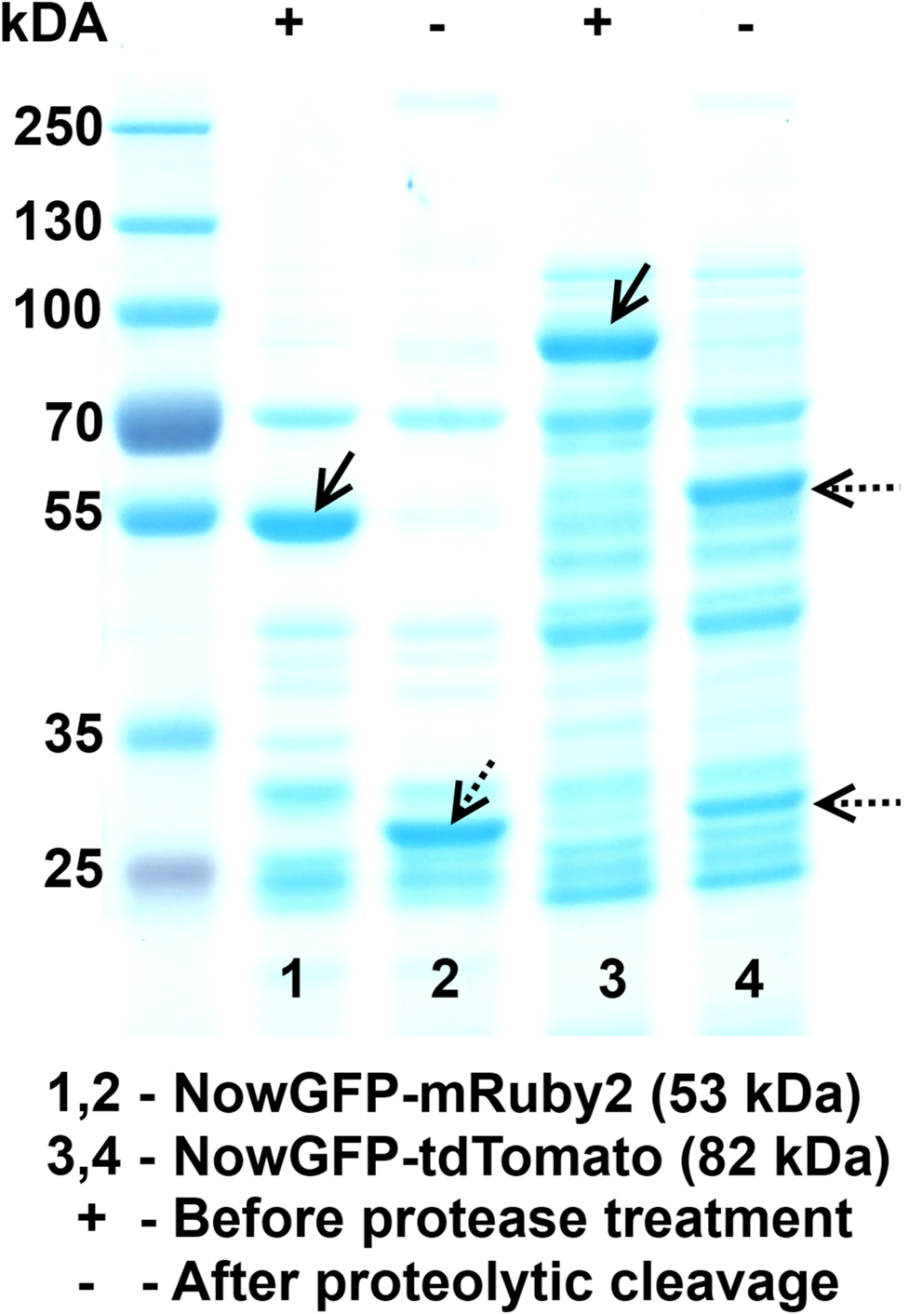
SDS PAGE displaying the proteolytic activity. The absence of fusion protein band and the presence of the cleaved protein band is visible from Lane 2 and 4 confirming proteolytic cleavage. The dotted arrows indicate the cleaved product and the straight arrow indicate the fusion protein band.

The results from SDS-PAGE further affirm that the change in fluorescence response on addition of thrombin is indeed due to the site-specific proteolytic cleavage by thrombin. The SDS-PAGE analysis (Fig 4) showed the cleaved protein bands at appropriate molecular weight. The fusion protein band was not detected on samples treated with thrombin. In samples without thrombin treatment, the fusion protein bands were evident while no visible bands were detected at molecular weights corresponding to the cleaved proteins. This confirms the proteolytic activity and affirms that the change in fluorescence response is solely due to the proteolytic activity.

**Fig 5:**
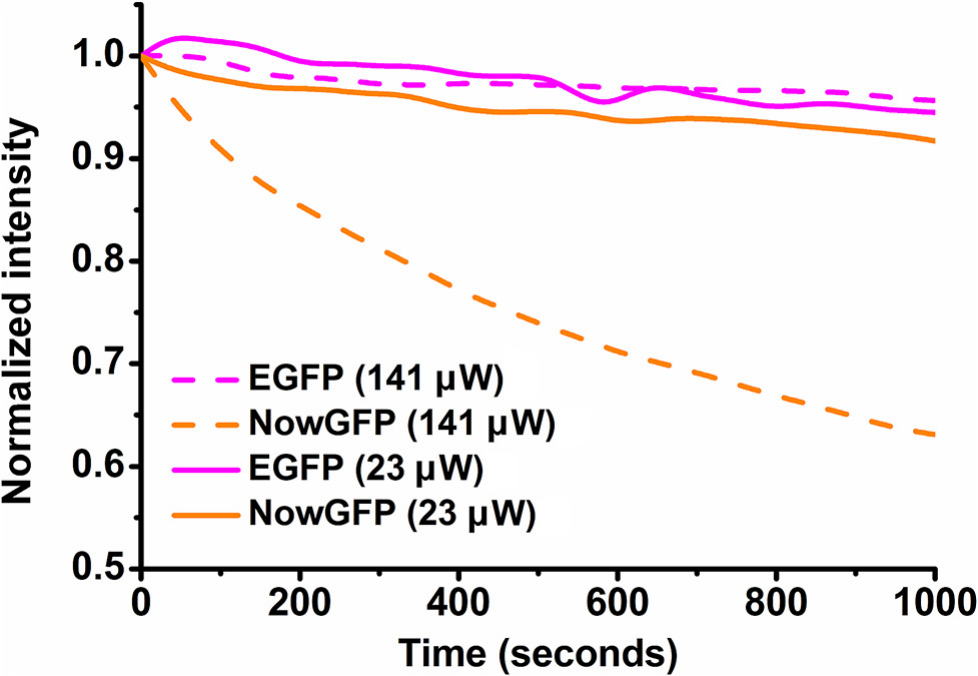
Photobleaching of NowGFP and EGFP in *E*. *coli* cells. Bacterial cells expressing the fluorescent proteins were excited at 488 nm laser, and the photobleaching was analyzed from images acquired using confocal microscope equipped with 63x oil objective. The difference in photobleaching of NowGFP and EGFP at different laser intensities (141 μW and 23 μW) can be observed from the figure.

One limitation for the use of NowGFP in intensity based FRET measurements is the higher photobleaching rate observed for NowGFP compared to EGFP in *in vivo* (Fig 5) and *in vitro* [18] photobleaching analysis. However, on reducing the laser intensity, the photobleaching rate of NowGFP has found to be significantly reduced and equivalent to EGFP photobleaching. It has also been shown that the NowGFP can be imaged at moderate light intensities (less than ~0.05 W/cm^2^) to obtain well resolved images in confocal mode [18]. Nevertheless, the photobleaching rate has to be taken into account for the use of NowGFP in intensity based measurements, especially for measurements involving long time and high intensity irradiation. However, this should not have an impact on fluorescence lifetime measurements as FLIM is generally performed in very low excitation light level which minimizes photobleaching [8,27]. Previous studies have revealed that there was no variation in fluorescence lifetime for fluorescent protein variants due to photobleaching from excitation light in FLIM [27,28]. Similarly, we have not observed any change in fluorescence lifetime of NowGFP in TCSPC and FLIM techniques during our experiments making NowGFP appropriate for fluorescence lifetime based studies.

### Intracellular measurements

To evaluate the FRET inside living cells, the constructs were expressed in *E*. *coli* cells. After overnight incubation, a bright fluorescence was observed in all the cultivations. Further incubation did not lead to noticeable difference in fluorescence which indicate the complete folding of the both the donor and acceptor fluorescent proteins. For intracellular studies, FRET was analyzed using FLIM by comparing the cells expressing donor alone with the cells expressing FRET pair. The decrease in the donor lifetime was selectively captured with fluorescence band pass filter (510/20 nm) which transmits only donor emission and blocks the emission of the acceptors. This was evident from FLIM of the acceptor alone which showed no emission of the red fluorescent protein. Further validation was obtained from the steady-state fluorescence spectrum of the purified mRuby2 or tdTomato as no emissions below 525 nm were detected.

**Fig 6:**
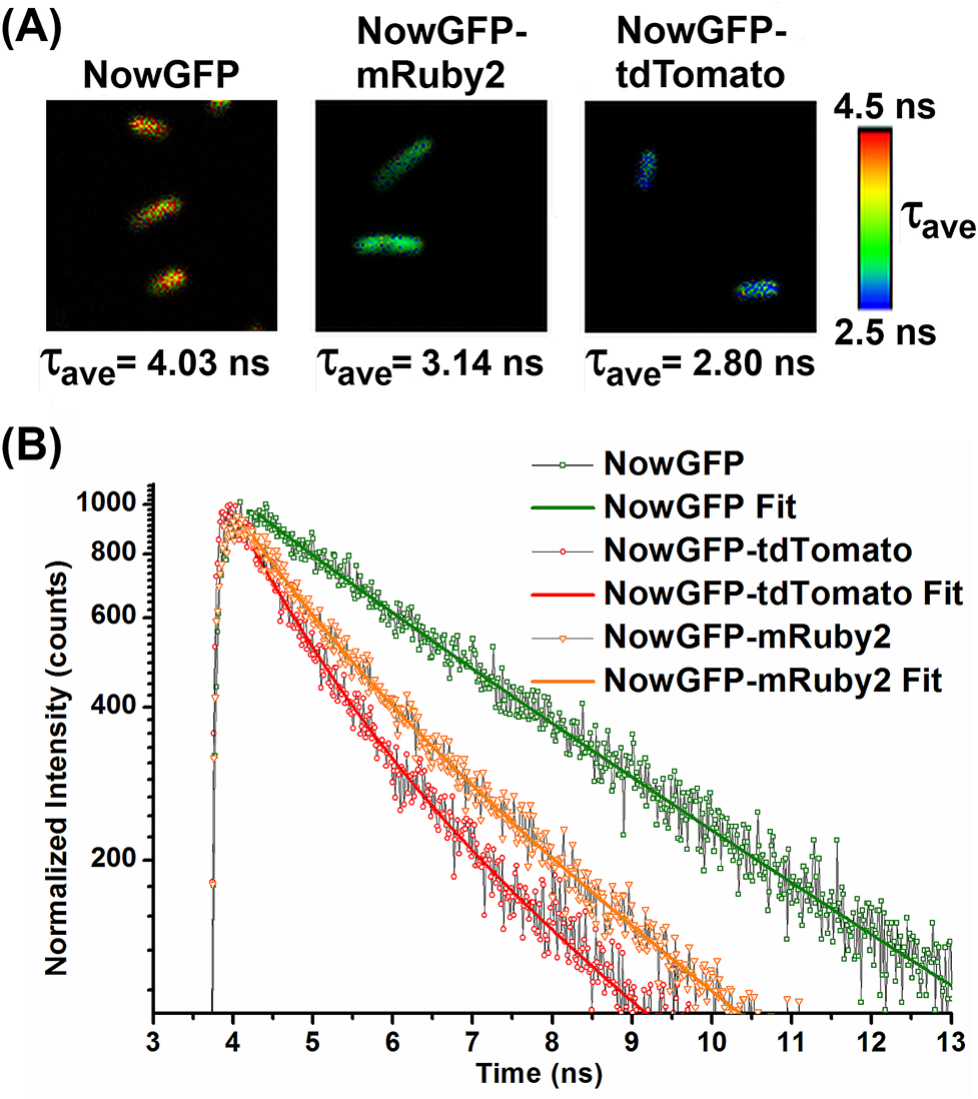
Intracellular FLIM of *E*. *coli* cells. *(A)* Fluorescence lifetime image of the cells displaying FRET. The cells are excited at 483 nm and the selective emission from the donor was monitored through band pass filter (510/20 nm). NowGFP is the cells expressing donor alone and the variation in lifetime as a result of FRET can be observed from the cells expressing the FRET pairs. The average lifetime is calculated from approximately 30 cells. Image size is 10 μm × 10 μm. *(B)* Fluorescence decay curve from the cells showing FRET. The decrease in the fluorescence lifetime due to FRET can be observed from the decay curve.

The high quantum yield and brightness of NowGFP enabled the use of low intensity excitation which helps to reduce problems related to the photobleaching of the fluorescent proteins. From the FLIM measurements, the *in vivo* fluorescence lifetime of NowGFP was observed to be 4.04 ns (Fig 6). In the studied FRET pairs, conspicuous difference in fluorescence lifetime of NowGFP was observed in the presence of an acceptor, even though the dynamic range was found to be reduced in the intracellular measurements in comparison with *in vitro* measurements. The fluorescence lifetime of NowGFP was reduced to 3.14 ns in NowGFP-mRuby2 FRET pair and 2.80 ns in NowGFP-tdTomato FRET pair (Table 3). Thus high *in vivo* dynamic ranges for the FRET pairs were recorded in comparison with previous FRET pairs having comparable linker lengths [28–31]. Based on the steady-state, TCSPC and FLIM measurements, the highest FRET efficiency was obtained with the NowGFP-tdTomato FRET pair.

**Table 3:** Fluorescence lifetime and FRET efficiency of the FRET pairs inside bacterial cells (From an average of approximately 35 individual cells, for each fluorescent protein/pair). E—FRET efficiency (calculated according to Eq 3). χ2—calculated standard weighted least squares to assess the goodness of the fit.

Fluorescent protein/pairs	τ_ave_	χ^2^	E
NowGFP (Donor alone)	4.04 ± 0.02	0.94	-
NowGFP-mRuby2	3.14 ± 0.01	0.93	0.22
NowGFP-tdTomato	2.80 ± 0.03	0.95	0.31

## Discussion

To develop novel FRET pairs with high dynamic range for biological applications, we exploited the new higher lifetime of NowGFP and the advantages of using red fluorescent protein variants in FRET pairs. Four variants of red fluorescent proteins were selected based on their fluorescent properties and spectral overlaps with NowGFP to generate the following FRET pairs, NowGFP-mOrange, NowGFP-mRuby2, NowGFP-TagRFP and NowGFP-tdTomato. The mOrange, mRuby2 and TagRFP are monomeric fluorescent proteins and have been previously reported as excellent acceptors for FRET [2,3,32]. The tdTomato is a tandem dimer (two fluorescent proteins coded in a single open reading frame), which is one of the brightest red fluorescent protein variant known and has proven to be an excellent FRET acceptor with GFP variants [20,33]. Other red fluorescent proteins such as mRFP and mCherry were not studied here as their emissions are very low to be detected above the donor emission tail causing challenges in ratiometric imaging [2]. The incorporated thrombin recognition site between the fluorescent proteins enabled us to study the changes in FRET by enzymatic activity. The physical separation of the FRET pairs increases the inter-chromophore distance and thus reduces FRET efficiency.

The high quantum yield of the donor, along with the high extinction coefficient (ε) of the selected acceptors (Table 1) is an advantage generating better FRET pairs [13]. The other main criteria that were considered for generating superior FRET pairs include reduction of undesirable overlap between donor and acceptor emissions, increased Förster distance, and FRET efficiency close to 0.5. The steady-state measurements revealed good separation between donor and acceptor emissions for the selected FRET pairs. The only exception was for NowGFP-mOrange FRET pair due to the fact that the emission of mOrange overlaps considerably with the one of NowGFP. The mRuby2 exhibited the largest donor-acceptor emission separation with NowGFP among the selected fluorescent protein variants making it a good candidate for FRET analysis with NowGFP as the donor.

The *R*_0_ value was determined from the spectral data. To the best of our knowledge, the *R*_0_ of NowGFP-tdTomato (6.57 nm) is the largest *R*_0_ value reported so far for any fluorescent protein based FRET pairs yet used in FRET applications or studies. The highest *R*_0_ value reported previously (6.3 nm) was for Clover-mRuby2 [2]. With the NowGFP-mRuby2 pair, the *R*_0_ (6.17 nm) recorded being the third highest value reported. The combination of high donor quantum yield, high extinction coefficients of the acceptors and large overlap integral has resulted in obtaining high *R*_0_ value for these FRET pairs. The high *R*_0_ value is important as the FRET is highly dependent on *R*_0_ and the FRET efficiency increases when *R*_0_ increases [2,34]. The higher *R*_0_ value of NowGFP-tdTomato and NowGFP-mRuby2 was reflected in the FRET measurement as these two FRET pairs showed higher FRET efficiency compared to the other FRET pairs analyzed. It should be also noted that the maximum FRET efficiency is limited by the physical size of fluorescent protein since the chromophore is located in the center of β barrel and FRET efficiency is distance dependent. The distance of closest approach between the chromophores of fluorescent proteins is ~ 3–3.1 nm and this occurs when the fluorescent domains are close together and in parallel orientation [26,35]. However, in a FRET sensor, the linker or sensing domain will further increase the distance between the FRET pairs and this reduces the FRET efficiency. Considering this factor, the FRET efficiency obtained for NowGFP-tdTomato and NowGFP-mRuby2 is reasonably high for fluorescent protein based FRET pairs and this can be attributed to its high *R*_0_ values.

The FRET efficiency and the distance between the fluorophores are related by a sigmoidal curve according to the Förster equation. The curve has the highest slope when the FRET efficiency is 0.5 and the interchromophore distance is close to *R*_0_. Due to this, FRET sensors operating with FRET efficiency close to or more than 0.5 exhibits large relative change in FRET efficiency with the change in interchromophore distance compared to the sensors which has FRET efficiency less than 0.5 [2]. Both NowGFP-mRuby and NowGFP-tdTomato have FRET efficiencies close to 0.5. This along with the high Förster radius of NowGFP-mRuby2 and NowGFP-tdTomato FRET pairs provides an explanation for the large change in the FRET ratio observed for these FRET pairs on proteolytic cleavage making it suitable FRET pairs for the design of FRET based sensors. One main concern of using tdTomato over other fluorescent protein variants stated previously is its bulkiness (54 kDa) as it is a tandem dimer [36–38]. Conversely, our results from enzymatic activity toward a FRET pair with the tdTomato showed that the bulkiness of tdTomato has not affected the kinetics of the proteolysis when comparing to the results obtained from smaller, monomeric variants. Similar results have previously been reported by using tdTomato in a FRET pair [20] which suggests that the use of tdTomato will not affect the biosensing property when it is used as a FRET acceptor in biosensor design. However in some fusions, tdTomato is reported to cause maturation issues and this has to be taken in to account on designing biosensors which involves complex structures or interactions [29]. Despite this, tdTomato is the brightest red variant, has a maturation half-time of one hour, used successfully for both N- and C-terminal fusions and in FRET sensors making it a good choice for an acceptor [29,33].

FRET evaluation in FLIM experiments relies only on measuring the donor lifetime in the presence and absence of acceptor, which is one of the most direct and robust ways to analyze FRET [29,32,39]. The large change in fluorescence lifetime denotes sensor with large change in FRET dynamic range. Our speculation that the higher lifetime of NowGFP could bring larger dynamic range for the sensor was verified from the *in vitro* fluorescence lifetime measurement which showed large reduction in the donor lifetime for NowGFP-tdTomato pair followed by NowGFP-mRuby FRET pair. The donor lifetime reduction observed in the *in vitro* FRET of NowGFP-tdTomato FRET pair was more than 2.6 times and this is the highest change reported so far for any fluorescent protein based FRET pairs. The larger separation between emission spectrum of the donor and acceptor was advantageous as no acceptor emission bleed through the monitoring filter making selective monitoring of the donor emission straightforward. Furthermore, the higher brightness of NowGFP enables low intensity excitation reducing photodegradation during the experiments. The large change in donor lifetime when undergoing FRET, along with the advantage of having large emission spectral separation and the higher brightness of NowGFP empowers NowGFP-tdTomato and NowGFP-mRuby2 to be used as superior FRET pairs for FLIM studies.

The intracellular FLIM analysis from *E*. *coli* cells demonstrated substantial difference in fluorescence lifetimes, when NowGFP is undergoing FRET with NowGFP-tdTomato exhibiting larger FRET similar to the results from *in vitro* measurements. However, a decrease in the fluorescent lifetime was observed for NowGFP (donor alone) in the intracellular measurements compared to the *in vitro* values, and this was reflected in the FRET dynamic range. The FRET dynamic range was found to be reduced in intracellular measurements compared to the *in vitro* measurements. The reduction in the lifetime in comparison with *in vitro* measurements can be attributed to the intracellular microenvironment which affects the fluorescence lifetime [40]. Furthermore, studies have shown that the change in the local microenvironment of the fluorophore can affect the donor lifetime and hence this must be considered when comparing the FRET lifetime in different organelles of a specimen [41–43]. Though the variation in fluorescence lifetime as a result of FRET is reduced compared to *in vitro* measurements, the *in vivo* fluorescence lifetime change of the FRET pair is still on higher side relative to the previously reported red fluorescent protein based FRET pairs [3,29,44]. The large change in FRET lifetime of the novel FRET pairs is beneficial in the development of improved FRET based sensors which switches between well-defined ON and OFF states.

Spectrally similar GFP variants, co-expressed in the same cell, have been reported to be separated based on fluorescence lifetime using FLIM [45]. Recently, NowGFP and EGFP signals from the same cell were shown to be separated based on fluorescence lifetime contrast [18]. Hence, we believe that the longer lifetime of the NowGFP-Ruby FRET pair can offer the possibility of FLIM with two different FRET-based biosensors in a single cell, when combined with another FRET pairs having shorter donor lifetime (for example TagGFP-TagRFP pair [3]).

## Conclusion

The novel FRET pairs reported here displayed improved FRET lifetime dynamic range and high FRET efficiency. Among the four FRET pairs analyzed, NowGFP-tdTomato and NowGFP-mRuby2 turned out to the superior FRET pairs making them excellent FRET pairs for FLIM and biosensors. Despite the higher brightness and quantum yield of NowGFP compared to EGFP, NowGFP bleaches faster than EGFP. However, NowGFP can be imaged under low light excitation and this reduces photobleaching. In addition, moving the excitation from Cyan (of traditional CFP-based FRET pairs) to green region will decrease phototoxicity issues in cells. The fluorescence spectrum of NowGFP [18] enables good spectral overlap and emission spectral separation with most of the red fluorescent protein variants. All these advantages combined with the longest reported fluorescence lifetime, makes NowGFP a good choice for a donor in FLIM-based FRET assays. NowGFP-tdTomato demonstrated the highest reported *R*_*0*_ value for any fluorescent protein based FRET pair used in biological studies and this was reflected in the FRET assay which showed wide FRET dynamic range in fluorescence lifetime measurements.

The new FRET pairs described will be a suitable choice for the new FRET based sensors in FLIM assays with improved dynamic range as well as to monitor protein interactions with high contrast. The detection at longer lifetimes also offers possibility for designing dual biosensors enabling detection of more than one protein interaction simultaneously from a single cell by FLIM.

## Supporting Information

S1 Table Primer designation and sequences used for generating FRET pairs. (DOCX)

S2 Table Primers and template with the PCR combinations used to create FRET pairs (in sequential order). (DOCX)

S1 Fig Amino acid sequence of NowGFP. The amino acid substitutions compared to mCerulean is marked in red inside the sequence and the substitutions are also mentioned in the bottom part of the figure
(TIF)

S2 Fig Full time window showing fluorescence decay and fits. (A) of the FRET pairs at monitoring wavelength of 515 ns along with the residues of fit (B). The quenching of the fluorescence lifetime as a result of FRET can be observed from the fluorescence lifetime decay of the FRET pairs.
(TIF)
