# Supplementary material for: Fluorescent Protein Based FRET Pairs with Improved Dynamic Range for Fluorescence Lifetime Measurements
Source: PLoS One. 2015 Aug 3;10(8):e0134436. doi: 10.1371/journal.pone.0134436 (PMC4523203; doi:10.1371/journal.pone.0134436)
Supplement: S1 Table — (DOCX) [file pone.0134436.s001.docx]

Table S1. Primer names sequences used for generating FRET pairs along with the primer functions

| **Primer name** | **Sequence (in 5’-3’ direction)** | **Primer function** |
| --- | --- | --- |
| Now-1 | CAATTGTGAGCGGATAACAATTTCACACAGAATTC | Binds to pQE-30 plasmid at its RBS (Used for amplifying NowGFP in NowGFP/pQE-30 vector) |
| Now-2 | CAGTGAGCCACCACCTGACTTGTACAGCTCGTCCGCGCCGA | Amplify NowGFP, Removes stop codon and creates GGGSL at 3’ end |
| Now-3 | AGAACCACGCGGAACCAGTGAGCCACCACCTGACTTGTAC | Extends the linker at 3’ end of NowGFP to GGGSLVPRGS |
| Or-1 | TCACTGGTTCCGCGTGGTTCTGTGAGCAAGGGCGAGGAGAATAAC | Amplify 5’ of mOrange and create overlap region SLVPRGS at 5’ end |
| Or-2 | GATATAAGCTTACTACTTGTACAGCTCGTCCA | Amplify 3’ of mOrange and creates 3' *Hind*III site at 3’ end |
| Tag-1 | TCACTGGTTCCGCGTGGTTCTATGGTGTCTAAGGGCGAAGAGCTGA | Amplify 5’ of TagGFP and create overlap region SLVPRGS at 5’ end |
| Tag-2 | TATAGAAGCTTATCAATTAAGTTTGTGCCCCAGT | Amplify 3’ of TagRFP and creates 3' *Hind*III site at 3’ end |
| Rub-1 | TCACTGGTTCCGCGTGGTTCTATGGTGTCTAAGGGCGAAGAGCTG | Amplify 5’ of mRuby2 and create overlap region SLVPRGS at 5’ end |
| Rub-2 | CTATAGAATAGGGCCCTCTAGAGAAGCTTACTTGT | Amplify 3’ of mRuby2 and creates 3' *Hind*III site at 3’ end |
| Tom-1 | TCACTGGTTCCGCGTGGTTCTATGGTGAGCAAGGGCGAGGAGGTCATC | Amplify 5’ of tdTomato and create overlap region SLVPRGS at 5’ end |
| Tom-2 | GTATGAAGCTTACTTGTACAGCTCGTCCATG | Amplify 3’ of tdTomato and creates 3' *Hind*III site at 3’ end |
