# Supplementary material for: Fluorescent Protein Based FRET Pairs with Improved Dynamic Range for Fluorescence Lifetime Measurements
Source: PLoS One. 2015 Aug 3;10(8):e0134436. doi: 10.1371/journal.pone.0134436 (PMC4523203; doi:10.1371/journal.pone.0134436)
Supplement: S2 Table — (DOCX) [file pone.0134436.s002.docx]

Table S2. Primers and template with the PCR reaction combinations to create FRET pairs (in sequential order)

| **PCR number** | **Primer (Sense)** | **Primer 2 (Antisense)** | **Template** |
| --- | --- | --- | --- |
| PCR-1 | Now-1 | Now-2 | NowGFP/pQE-30 |
| PCR-2 | Now-1 | Now-3 | PCR-1 |
| **NowGFP-mOrange FRET pair** | | | |
| PCR-3 | Or-1 | Or-2 | pET mOrange LIC cloning vector (u-mOrange) * |
| PCR 4 | Now-1 | Or-2 | PCR-2 and PCR-3 |
| **NowGFP-TagRFP FRET pair** | | | |
| PCR-5 | Tag-1 | Tag-2 | pTagRFP-N vector (Evrogen, Cat.# FP142) |
| PCR-6 | Now-1 | Tag-2 | PCR-2 and PCR-5 |
| **NowGFP-mRuby2 FRET pair** | | | |
| PCR-7 | Rub-1 | Rub-2 | pcDNA3.1-Clover-mRuby2 † |
| PCR-8 | Now-1 | Rub-2 | PCR-2 and PCR-7 |
| **NowGFP-tdTomato FRET pair** | | | |
| PCR-9 | Tom-1 | Tom-2 | pmAmetrine-DEVD-tdTomato‡ |
| PCR-10 | Now-1 | Tom-2 | PCR-2 and PCR-9 |

* Addgene plasmid # 29770

† Addgene Plasmid #49089

‡ Addgene plasmid 18879
