# Supplementary figures and images for: Fluorescent Protein Based FRET Pairs with Improved Dynamic Range for Fluorescence Lifetime Measurements
Source: PLoS One. 2015 Aug 3;10(8):e0134436. doi: 10.1371/journal.pone.0134436 (PMC4523203; doi:10.1371/journal.pone.0134436)

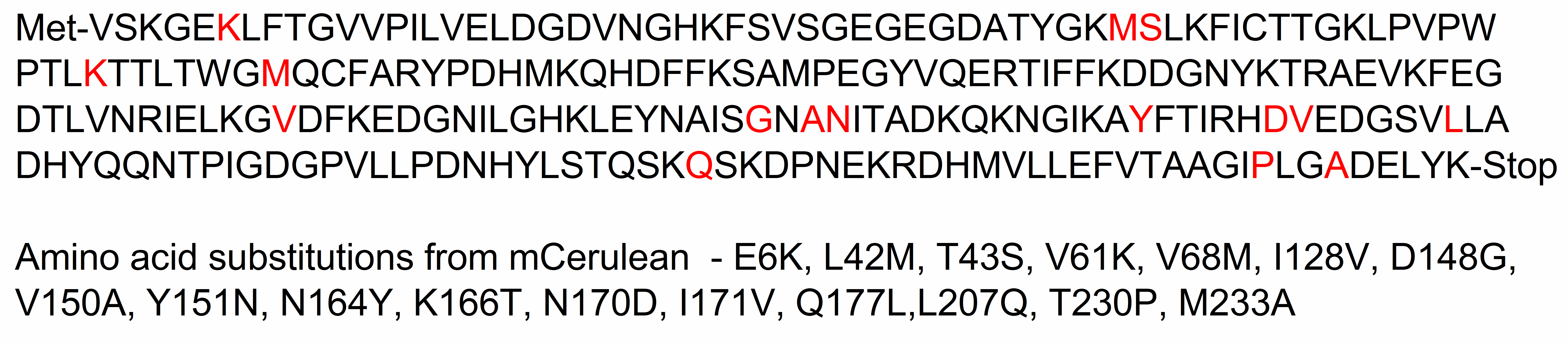

Supplement: S1 Fig — The amino acid substitutions compared to mCerulean is marked in red inside the sequence and the substitutions are also mentioned in the bottom part of the figure (TIF) [file pone.0134436.s003.tif]

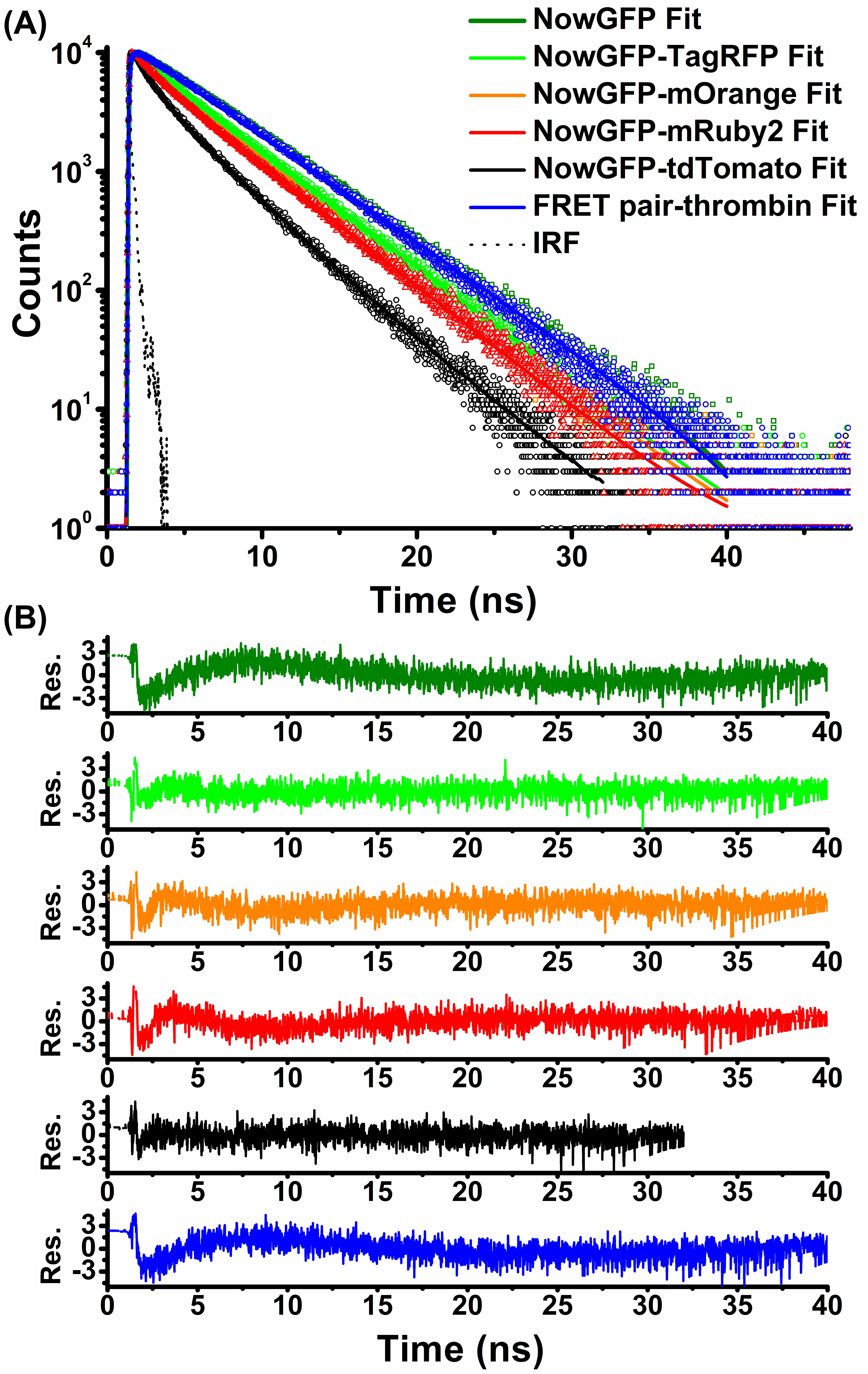

Supplement: S2 Fig — (A) of the FRET pairs at monitoring wavelength of 515 ns along with the residues of fit (B). The quenching of the fluorescence lifetime as a result of FRET can be observed from the fluorescence lifetime decay of the FRET pairs. (TIF) [file pone.0134436.s004.tif]
